# Analgesic Opioid Misuse and Opioid Use Disorder among Patients with Chronic Non-Cancer Pain and Prescribed Opioids in a Pain Centre in France

**DOI:** 10.3390/ijerph18042097

**Published:** 2021-02-21

**Authors:** Morgane Guillou-Landreat, Bertrand Quinio, Jean Yves Le Reste, Delphine Le Goff, Jérôme Fonsecca, Marie Grall-Bronnec, Antoine Dany

**Affiliations:** 1 INSERM UMR 1246, SPHERE, Methods in Patient-Centered Outcomes and Health Research, Nantes and Tours Universities, 44000 Nantes, France; marie.bronnec@chu-nantes.fr; 2 EA 7479 SPURBO, BREST University, 29200 Brest, France; lerestejy@gmail.com (J.Y.L.R.); docteurdlegoff@gmail.com (D.L.G.); jeromefonseca@hotmail.com (J.F.); 3 HUGOPSY Network, 35000 Rennes, France; 4 CHRU, Hopital de la Cavale Blanche, 29200 Brest, France; bertrand.quinio@chu-brest.fr; 5 CHU Nantes, Addictology and Psychiatry Department, 44000 Nantes, France

**Keywords:** prescribed opioids, opioid misuse, opioid use disorder, chronic non-cancer pain

## Abstract

(1) Background: Chronic non-cancer pain (CNCP) remains a public health challenge around the world. Opioids (PO) have been increasingly used in the treatment of CNCP in the last 20 years. This study aimed to assess the prevalence of opioid misuse and prescribed-opioid use disorder (p-OUD) among patients with CNCP in a pain centre in France, and to analyse risk factors for moderate or severe p-OUD. (2) Method: A cross-sectional study was conducted, including patients consulting for pain management in the pain centre of Brest University Hospital. A self-questionnaire was administered (sociodemographic data, medical data, PO misuse, and p-OUD according the Diagnostic and Statistical Manual of Mental Disorders 5 (DSM 5) criteria). Descriptive, univariate, and multivariate analyses were conducted, together with a principal component analysis, in order to identify factors associated with p-OUD. (3) Results: In total, 115 patients were included, the majority of whom were women, with a mean age of 52 years old [18–82]; 64.3% (*n* = 74) had a current prescription for opioid analgesics (weak or strong). In this group, 56.7% (*n* = 42) had no or only mild p-OUD and 43.3% (*n* = 32) had current moderate or severe p-OUD. Patients with moderate or severe p-OUD were more likely to have a current antidepressant prescription, to have had psychotherapy, to currently use strong opioids and oxycodone, and to report taking more frequent doses than prescribed and feeling dependent. (4) Conclusions: We showed that the prevalence of current moderate/severe p-OUD concerned 43.3% of the patients with a CNCP seeking treatment in a pain centre. According to these results, several measures are relevant in managing p-OUD among patients with CNCP.

## 1. Introduction

Chronic non-cancer pain (CNCP) includes any painful ailment that lasts for 3 months or more and is not related to a malignancy [1]. CNCP is a destructive phenomenon, with potentially overwhelming damage (physical, psychological, familial, and economic) [2], and currently remains a public health challenge around the world [3]. In Europe, the prevalence of CNCP is estimated to be 19% [4], and in France, in the general population, chronic pain affects 31.7% of the adult population [5]. In the last 20 years, opioids have been widely used in the treatment of acute and cancer pain, and also for chronic non-cancer pain [6,7]. An increasing use of opioids has been observed in the last decade [3]. In the USA, the “opioid epidemic” is characterised by aggressive prescribing practices among medical practitioners [8] and a rapid increase in the prescription of opioids, which is linked to the increase in opioid overdoses [9]. In Europe, an increase in strong opioid prescriptions has also occurred, with most prescriptions being for CNCP [3]. In the UK, it has been estimated that nearly one million people are using some form of opioid [10]. This is a particular concern in France, as prescription opioid (PO) use between 2004 and 2017 at least doubled, and oxycodone use increased particularly markedly, alongside a non-trivial increase in opioid-related morbidity and mortality [11]. The prevalence of a long-term prescription of opioids for CNCP is estimated to be 1.9% and the prevalence of strong opioid use is estimated to be 1.1% [11].

There is poor evidence that the use of opioids for CNCP significantly reduces pain in the long term, and no conclusive evidence of improvement in the quality of life or functioning has been produced [12,13,14]. Long-term opioid use has also been associated with hyperalgesia and impairment of the health status, leading to increased disability, and opioid misuse and dependence [15,16,17,18,19,20,21]. PO misuse is defined as the use of opioids contrary to the prescriber’s directions [6]. Opioid misuse contributes to the increase in opioid use disorders and opioid-related deaths [20]. Opioid use disorder (OUD) is characterised by the persistent use of opioids, despite the adverse consequences of their use, alongside impaired control, the compulsive use of opioids and cravings [6,22]. 

Despite the high prevalence of CNCP and the frequent prescription of opioids, data on the prevalence, risk factors, clinical assessment and management of CNCP and opioid misuse and prescription OUD (p-OUD) is still sparse, particularly in France [6]. A recent study highlighted a high prevalence of 76.9% of misuse of PO by adults with CNCP in a sample of 52 patients evaluated during hospitalization, where 52% fulfilled at least six Diagnostic and Statistical Manual of Mental Disorders 5 (DSM 5) criteria for p-OUD [23]. However, this fairly severe, hospitalised sample was quite small. In addition, the clinical variables associated with misuse and OUD were not explored. It is crucial to analyse clinical factors associated with the risk of p-OUD, as they could help clinicians managing CNCP and PO analgesics, for instance, general practitioners, to detect p-OUD early and to adapt their management strategies.

This study aimed to assess the prevalence of current and lifetime opioid misuse and prescription-opioid use disorders (p-OUD) among patients with CNCP in a pain centre in France, and to analyse risk factors and risk indicators for moderate or severe p-OUD. 

## 2. Materials and Methods

### 2.1. Method 

A cross-sectional study was conducted, including patients consulting for pain management in the pain centre (CETD) of Brest University Hospital.

### 2.2. Population 

Consecutive patients were recruited over a 3-month period from June to September 2016. Patients included were French speakers aged at least 18, which were referred for the first time to the pain centre for chronic non-cancer pain with a current or lifetime use of a PO (at least one month). All patients provided signed informed consent. 

Patients with pain caused by a current cancer or unable to provide informed consent were excluded. 

### 2.3. Data Collection

A self-administered questionnaire was developed by the study scientific committee, which included physicians specialised in pain and addictive disorders, general practitioners (GPs), and methodologists. The questionnaire was previously tested on a small sample of patients. 

All patients referred by their general practitioner to the pain centre in the inclusion period were sent the questionnaire to identify patients with opioid misuse and p-OUD. The patients completed questionnaires at home and returned them at the first visit. 

The following data were collected in the self-administered questionnaire. 

Socio-demographic data: Gender, date of birth, number and age of children, and marital and professional status. 

Medical data: Personal medical and surgical history, lifetime history of pain management (current and past use of analgesics: Nonopioids, such as NSAIDs and paracetamol; weak opioids, such as codeine, tramadol, etc.; and strong opioids, such as morphine, oxycodone, etc.; non-analgesic drugs: Antidepressants, anxiolytics, antipsychotics, etc.; and non-drug therapies). 

Opioid misuse variables according to the items in the Prescription Opioid Misuse index (POMI) were collected, but questions regarding medical nomadism were excluded, since patients completed the questionnaire before meeting physicians in the pain centre [24]: The use of higher doses than prescribed; more frequent use than prescribed; and the search for effects other than pain relief. Questions related to feelings of being dependent on PO were added, as well as questions regarding the sharing of analgesic PO with relatives. 

Opioid use disorders (OUD): Current and lifetime prescribed opioid use disorders (p-OUDs) according to the DSM 5 (Diagnostic and Statistical Manual of Mental Disorders) criteria were collected by the questionnaire [22]. This method has been used previously. Each item is expressed in the form of a closed-ended question, validated by the scientific committee [25]. All the DSM 5 criteria were collected [22] to gather information on OUD. For the diagnosis of p-OUD (prescribed-opioid use disorders), tolerance and withdrawal were excluded. The rationale for this was that these symptoms are likely to be iatrogenic, rather than psycho-pathogenic, in a therapeutic context [26,27]. The cut-off criteria for p-OUD were as follows: 2–3 symptoms for mild p-OUD; 4–5 symptoms for moderate p-OUD; and 6 or more symptoms for severe p-OUD. The moderate and severe p-OUD were combined for the comparative analysis, as there was a good correspondence between the DSM IV addiction diagnosis and moderate/severe OUD [28]. 

### 2.4. Statistical Analysis

Data were gathered and anonymised in an Excel file. Statistical analyses were performed using R software version 3.5.3. Type 1 error was set at 5%.

A descriptive analysis was performed to describe the socio-demographic data of the sample and relevant medical information, and in particular, to estimate the prevalence of p-OUD. 

Univariate and multivariate analyses were conducted to determine which variables were associated with having either moderate or severe p-OUD. Only variables that were significantly associated with moderate or severe p-OUD in the univariate analyses were included in the multivariate analysis. A stepwise backward elimination algorithm was used to eliminate variables that provided little information in the multivariate model. 

A principal component analysis was performed to identify whether there were highly correlated sets of variables among variables that were significant in the univariate analyses. The objective was to explore redundancy amongst the explanatory variables for p-OUD.

## 3. Results

### 3.1. Description of the Population

**Figure 1:**
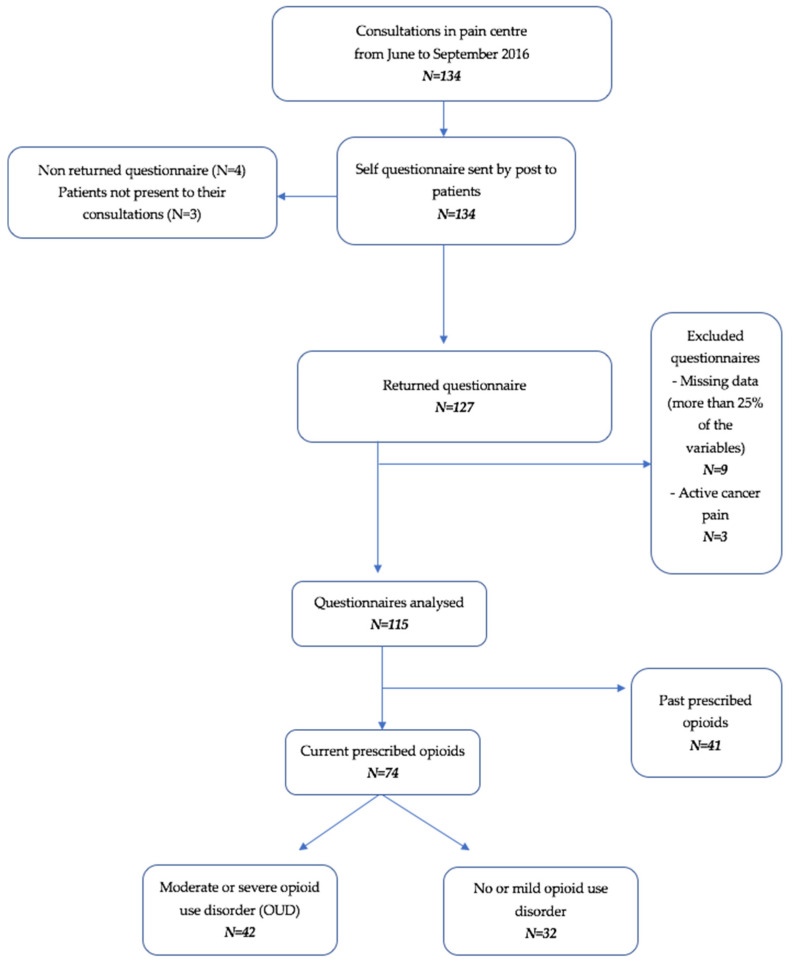
Flowchart of the study.

During the 3-month study, 134 patients were referred to the pain centre, and 115 patients were included in the present study (Figure 1). The mean age was 52 years [18–82]. In the sample, 61% (*n* = 71) were women, 62.6% had a marital status (*n* = 72), 54.8% were currently working (*n* = 63), 24.3% (*n* = 28) were retired and 20.8% (*n* = 24) were unemployed. Additionally, 27.8% (*n* = 32) were on sick leave, among whom 10.4% were on occupational sick leave or had an occupational illness. 

Descriptive characteristics of the population are presented in Table 1. 

#### 3.1.1. Opioid Misuse and Opioid Use Disorder 

Characteristics of opioid misuse behaviours are presented in Table 2. 

#### 3.1.2. Current and Lifetime p-OUD

In the sample, 64.3% (*n* = 74) had a current prescription for opioid analgesics (weak or strong). In this group, 56.7% (*n* = 42) had no or only mild p-OUD and 43.3% (*n* = 32) had current moderate or severe p-OUD. In the group of those meeting the DSM 5 criteria for p-OUD (*n* = 50), 36% (*n* = 18) had mild p-OUD, 34% (*n* = 17) had moderate p-OUD and 30% (*n* = 15) had severe p-OUD. 

For lifetime p-OUD, 40% (*N* = 46) of the sample were classified as having either moderate or severe lifetime p-OUD. 

The frequencies of positive criteria among the 11 DSM 5 criteria for the diagnosis of OUD are presented in Table 3. The four criteria that were the most frequently positive were a persistent desire or unsuccessful efforts to cut down or control PO use (37.4%, *n* = 43); recurrent PO use resulting in failure to fulfil major role obligations at work, school or home (36.5%, *n* = 42); continuing use despite knowledge of having a persistent or recurrent physical or psychological problem that is likely to have been caused or exacerbated by PO (34.8%, *n* = 40); and continuing PO use despite having persistent or recurrent social or interpersonal problems caused or exacerbated by the effects of PO (30.4%; *n* = 35). 

### 3.2. Comparative Analysis 

Patients with moderate or severe p-OUD were compared to patients with no or mild p-OUD (0 to 3 positive criteria). 

#### 3.2.1. Univariate Analyses

The results of the univariate analysis are presented in Table 4. For categorical variables, the *p*-value is associated with a Fisher exact test. For quantitative variables, the *p*-value is associated with a Student *t* test (or a Mann–Whitney test for complex distributions).

#### 3.2.2. Multivariate Analysis

The multivariate analysis with a backward elimination algorithm retained four variables that were all significantly associated with moderate or severe OUD (Table 5). The Hosmer–Lemeshow test did not detect any significant misfit in the resulting statistical model (*p* = 0.986, χ^2^ = 1.807, df = 8). 

Patients with moderate or severe p-OUD were more likely to have a current antidepressant prescription, to have had psychotherapy, to currently use strong opioids and oxycodone, and to report taking more frequent doses than prescribed and feeling dependent. 

### 3.3. Principal Component Analysis 

A principal component analysis was performed using variables that were significantly associated with p-OUD in the univariate analysis. The scree plot analysis and a parallel analysis retained four factors. The positions of patients and variables relative to the two main components are shown in Table 6.

Three factors were retained on the basis of the results from both the scree plot analysis and the parallel analysis. The most relevant factors are presented in Table 6: Factor 1 “Inadequate analgesic treatment” corresponded to a history of neurological disorders, past use of antipsychotics and past and current use of strong opioids; factor 2 “psychosocial vulnerability” corresponded to sick leave, psychotherapy and hypnosis; and the third factor “psychiatric and addictive vulnerability” corresponded to a history of psychiatric disorders, a history of addictive disorders and current antidepressant use. 

## 4. Discussion

### 4.1. A High Prevalence of Moderate or Severe Prescribed p-OUD

In the present study, 43.3% of CNCP patients referred for the first time to a specialized pain center reported current moderate or severe p-OUD. Among those meeting the p-OUD criteria, 34% had moderate p-OUD and 30% had severe p-OUD. The proportion of severe patients is smaller in our study than in a recent French study led by Eiden et al. (52%) [23], but they interviewed a sample of inpatients, while our sample was ambulatory, and the physicians in Eiden’s study assessed on the basis of the DSM 5 criteria, while we used a self-administered questionnaire, which could involve an underestimation of the disorders by patients. Regarding the international data, our prevalence is higher than previous findings from the USA, Australia [28,29,30] and in Germany. Indeed, a similar study was conducted there in 2018, and the authors found a prevalence of 26.5% for p-OUD [31]; but their population was older with a mean age of 61.8 years, compared to 52 years in our study, and young age is associated with p-OUD [31,32]. In addition, their recruitment took place in outpatient pain specialist units, whereas our recruitment was implemented in a university hospital. Our sample combined many CNCP severity factors, and risk factors for PO misuse and p-OUD [6,33]. The median duration of pain was 91 months, 44.8% had previously used strong PO in their lifetime, and 26.9% were still being treated with strong PO. The proportion with a psychiatric history was also high: 29.4% had a lifetime history of psychiatric illness, and 9% had a history of addictive disorders. 

Regarding the DSM 5 OUD criteria frequencies, the most frequent were the persistent desire to stop opioids (37.4%), the continuing use of opioids despite the psychological or physical damage induced or exacerbated by opioids (34.8%) and the failure to fulfil obligations due to opioid use (36.5%). This perceived damage in the daily life is coherent with the deterioration in quality of life described with long-term use of analgesic opioids [34]. Independently from the diagnosis of p-OUD, tolerance and withdrawal, not considered specific to p-OUD in the case of PO, were surprisingly poorly represented, at respectively 26.1% and 26.9% in our population, since the long-term use of PO could lead to these pharmacological phenomena. 

The patients also reported PO misuse: 16.5% of the sample did not always or never followed the medical prescriptions of PO, 15.6% reported that they used PO to obtain other effects than analgesia, 13.5% were using higher doses, and 10.4% reported a more frequent use than that provided for in the prescription. We also found that the use of more frequent doses of PO was significantly associated with moderate or severe p-OUD in the multivariate analysis (OR=5.36). 

Further to this, the feeling of being dependent on PO (OR = 3.7) was significantly associated with p-OUD in the multivariate analysis. This could reflect the need to implicate and inform patients before PO initiation on the risks of misuse and dependence [33]. Previous studies have highlighted the importance for prescribers of controlling for signs that could reflect PO misuse or dependence, such as a rapid increase in dose without clinical explanation, non-compliance with the prescription, systematic refusal to consider other treatments under various pretexts, and resort multiple prescribers, alongside tolerance phenomena or withdrawal symptoms [33]. None of these variables involve the patients’ feelings. Available tools that evaluate PO misuse (POMI) [24] and dependence (SOAPP-R) [35,36] evaluate patient behaviours but do not consider the patients’ feelings. The present study showed that, in line with a patient-centered approach, the question about the patients’ feelings of being dependent could be interesting, as this was significantly associated to the moderate or severe p-OUD. 

### 4.2. Factors Associated with p-OUD 

Factors significantly associated with p-OUD in the multivariate analysis were first related to pharmacological variables: current prescription of strong opioids (OR = 0.28), and current use of oxycodone (OR = 6.17) were both significantly associated with moderate or severe p-OUD. This could reflect the greater pharmacological addictive potential of strong POs, and more particularly oxycodone in this study [37]. In the factorial analysis (PCA), the two variables “past use of strong opioids (PO)” and “current use of strong opioids (PO)”, probably reflecting a long or lifetime history of strong opioid use, were related to a factor defined as the “Inadequate analgesic treatment factor”. The variable “neurological history” and past use of antipsychotics also contributed to this factor. The association between a history of neurological disease and current/past use of strong opioids could reflect a lack of training for physicians, who may consider the WHO levels for analgesics rather than characterising the type of pain and an adaptation of the treatment to the type and origin of pain, as recommended [34]. Our results are in accordance with conclusions from previous studies warning that strong PO should not be used in CNCP, particularly in presence of neurological pain such as fibromyalgia, chronic headache or lower back and neuropathic pain, because they are not efficient and are liable to damage quality of life or produce side effects [34]. It can be suggested that the association between a history of neurological disease and antipsychotic treatment could be explained by the use of antipsychotics for neurological pain, which is also an unsuitable prescription, as this off-label use entails a high risk of side effects [38]. The high lifetime (48.7%) and current (32.1%) prevalence of antipsychotic prescriptions found in the present study supports this hypothesis. 

The second factor identified in the factorial analysis, was the factor termed “psychosocial vulnerability”. This factor combined three variables: the first was being on sick leave, which was associated with having received psychotherapy, and hypnosis. Sick leave could reflect difficulties regarding employment, and probably difficult financial conditions. It could contribute to greater environmental vulnerability towards p-OUD [33]. One hypothesis for this association is that psychotherapy and hypnosis could reflect psychosocial support to face up to daily life difficulties as a result of sick leave. In the multivariate analysis, psychotherapy was statistically associated with p-OUD (OR = 4.35). One may also suppose that it could reflect depression-like symptoms, it could be either a vulnerability of the individual that promotes higher opioid use, or a consequence of opioid use in a vulnerable population, or independent of opioid use. The statistical association between these non-pharmacological interventions and p-OUD was surprising. But patients referred to the pain centre are likely to have complex disorders, to be dissatisfied with their treatment and to have previously experienced several pharmacological treatments. In the sample, the use of non-pharmacological treatments, recommended for the management of CNCP [34], was low. Only 14.8% had used hypnosis, 18.3% had psychotherapy and 18.2% relaxation therapy. We therefore hypothesize that the use of psychotherapy and hypnosis could reflect a last resort to relieve CNCP among the most severe treatment-resistant patients. Both could be used to help with p-OUD management, opioid withdrawal and therefore contribute to detecting patients with opioid misuse or p-OUD [39]. However, behavioural and mind-body interventions, including hypnosis, have proved their efficacy in the relief of CNCP (Cognitive Behavioural Therapy (CBT), mindfulness etc.) [39,40]. There is growing interest in this topic in the literature. Several studies have recently been published aiming to compare the efficacy of behavioural interventions for the management of CNCP [41,42]. These behavioural interventions, including hypnosis, are still rarely used in clinical practice, as shown in our study, and few physicians or nurses are being trained, especially in France [43]. Eilender et al. suggested that psychotherapeutic interventions for concurrent pain and substance misuse using mindfulness or CBT approaches could reduce pain severity and opioid misuse [44]. In addition, hypnosis could be a first-intention treatment for CNCP and precede pharmacological treatment [39,44].

The third factor, “psychiatric and addictive vulnerability” combines the current prescription of antidepressants, which was also significantly associated with p-OUD (OR=7.14) in the multivariate analysis, with the variables “history of addictive disorders” and “history of psychiatric disorders”. According to previous reviews, CNCP patients with a past or present history of substance use disorder and a psychiatric history, had a greater risk of PO misuse and dependence [6,45,46,47,48]. A history of psychiatric or addictive disorders is not always that easy to collect in daily practice, but in this study it was shown that a current antidepressant prescription was a good indicator of psychiatric and addictive vulnerability, and associated with a higher risk of p-OUD. 

### 4.3. Strengths and Limitation

This study has several limitations. It was a single-centre study, so that results cannot be generalized. The data was self-reported, while DSM-5 criteria assessed by a physician would have been preferable. The setting could influence patients, constituting a social desirability bias, but this method has already been applied in previous studies [25,31]. The high response rate, given the controversial topic, suggests that using self-report was a consistent approach. This method offered patients full anonymity and gave no disincentive for being honest, reducing these biases. However, we cannot rule out that some questions could have been misinterpreted. Positive responses to the DSM-5 criteria could be interpreted as a result of insufficient pain therapy rather than p-OUD, suggesting a confusion bias.

Strengths of the present study include firstly the fact that studies on OUD among patients with CNCP in a specialized centre are sparse, particularly in Europe. Secondly, this study collected clinical data related to pain and also to addictive disorders and their risk factors. To our knowledge, it is the first in France to follow this method. Finally, the sample is large, compared to the existing literature, even if results need to be confirmed in a multi-centre study.

### 4.4. Implications

In a therapeutic perspective, the high prevalence of moderate/severe OUD in this sample of patients with CNCP highlights the need to strengthen interdisciplinary communication, and to develop the multidisciplinary management of p-OUD, including pain physicians and physicians specialized in addictive disorders, as recently underlined by an international expert consensus [49]. Further to this, Volkow et al. recently emphasized the need to expand access to medication for p-OUDs to prevent fatalities and facilitate recovery of patients presenting p-OUD [37]. 

In a preventive perspective, improving PO practices for pain management is necessary [37]. Evidence suggests that most PO are prescribed in primary-care settings and the management of these prescriptions can become an issue for some primary-care physicians [50,51]. GPs, with their wealth of information about their patients and their background [52] have an important role to play in prevention, by identifying patients with CNCP associated with p-OUD [53]. We have shown that moderate/severe prescribed OUD was associated with three factors. Two concerned patient vulnerabilities: “psychiatric and addictive vulnerability”, and “psychosocial vulnerability”. These factors should be taken in account in a preventive approach. The third factor concerned pharmacological strategies and “inadequate analgesic treatment”. Regarding pharmacology, issues of PO in our study were mostly non-compliant with the recommendations. Further to this, few included patients had received non-pharmacological interventions, such as psychological support, hypnosis or relaxation. These results probably reflect the lack of stepped pain treatment in CNCP management and the failure to follow the current recommendations [54]. Harm-reduction strategies and primary care-led treatment models should be developed, including education on pain management to reduce opioid prescribing, and including non-pharmacological treatment and patient psycho-education. The implication of GPs is also essential when an OUD occurs, to provide access to medication for OUD [55]. Patients with a p-OUD are less likely to perceive themselves as dependent, they may fear the stigma of being an “addict” and they will thus be less likely to resort to specialized addictive disorder services than patients with addiction to illicit opioids [6,33,56]. 

## 5. Conclusions

In this study, we have shown that the prevalence of current moderate/severe p-OUD concerned 43.3% of the patients with a CNCP seeking treatment in a pain centre. We evidenced that factors correlated with the risk of p-OUD in this population concerned individual and environmental vulnerabilities and also factors linked to therapeutic strategies. According to these results, several measures are relevant in managing p-OUD among patients with CNCP, combining specialized care including pain and addiction treatment, and prevention measures in primary care. Primary care-based interventions could be developed targeting psycho-education of patients with CNCP along with non-pharmacological interventions such as hypnosis or CBT, patient information on prescribed opioid side-effects, including dependence risks, and an evaluation of pain treatment management centred on patients’ feelings and quality-of-life. The implementation of these measures in specialized and primary care in collaboration with physicians specialized in addictive disorders and chronic pain management could help to reduce p-OUD among patients with CNCP.

## Figures and Tables

**Table 1:** Characteristics of the population.

Variables	Mean [Min–Max]
**Duration of pain and current analgesic treatment**	
Duration of pain (months)	91 [3–468]
Current treatment duration (months)	19 [1–41]
**Medical/psychiatric lifetime history**	**% (*n*)**
Muscular skeletal disorders	77.4 (89)
Surgical (pain location)	44.3 (51)
Cardiovascular diseases	32.1 (37)
Digestive/urological/gynaecological	49.5 (57)
Neurological	18.2 (21)
Respiratory	18.2 (21)
Genetic/immunological/endocrine	14.8 (17)
Psychiatric	29.5 (35)
Addictive disorders	9 (10)
**Current use of analgesics**	
Non-opioid analgesics	60.8 (70)
Weak opioids	47.8 (55)
Strong opioids	26.9 (31)
**Current use of concomitant treatment**	
Antipsychotics	32.1 (37)
Antidepressants	18.2 (21)
Anxiolytics	32.1 (37)
Hypnotics	20.9 (24)
Others	49.5 (57)
**Past use of analgesics**	
Non-opioid analgesics	93 (107)
Weak opioids	71.3 (82)
Strong opioids	44.8 (52)
**Past concomitant treatment**	
Antipsychotics	48.7 (56)
Antidepressants	37.4 (43)
Anxiolytics	20.0 (23)
Hypnotics	8.7 (10)
**Non-pharmacological pain treatment**	
Physiotherapy	56.5 (65)
Psychotherapy	18.3 (21)
Acupuncture	24.3 (28)
Osteopathy	29.5 (34)
Transcutaneous electrical nerve stimulation (TENS)	16.5 (19)
Hypnosis	14.8 (17)
Relaxation	18.2 (21)

**Table 2:** Characteristics of opioid misuse.

Misuse Variables	% (N) *n* = 115
**Compliance with Prescription**	
“Never” or “not always”	16.5 (19)
“Most of the time” or “always”	74.8 (86)
Unknown	8.7 (10)
Higher opioid doses than prescribed	13.9 (16)
Higher frequencies of opioid intake than prescribed	10.4 (12)
Different route of administration than prescribed	2/6 (3)
Non-antalgic use	15.6 (18)
Sharing treatments with others	4.3 (5)
Feelings of being dependent on analgesic PO *	38.2(44)

* PO: Prescribed Opioids.

**Table 3:** Frequency of the Diagnostic and Statistical Manual of Mental Disorders 5 (DSM 5) criteria for opioid use disorder (OUD) in the population (*n* = 115).

DSM 5 Criteria	% (*n*)
Opioids taken in larger amounts or over a longer period of time than intended.	25.2 (29)
Persistent desire or unsuccessful efforts to cut down or control opioid use.	37.4(43)
A great deal of time is spent in activities required to obtain the opioid, use the opioid, or recover from its effects	27.8 (32)
Craving or a strong desire to use opioids	6.1 (7)
Recurrent opioid use resulting in failure to fulfil major role obligations at work, school or home	36.5 (42)
Continuing opioid use despite having persistent or recurrent social or interpersonal problems caused or exacerbated by the effects of opioids	30.4 (35)
Important social, occupational or recreational activities are given up or reduced because of opioid use.	27.8 (32)
Recurrent opioid use in situations in which it is physically hazardous	20.8(24)
Continuing use despite knowledge of having a persistent or recurrent physical or psychological problem that is likely to have been caused or exacerbated by opioids	34.8(40)
Tolerance *	26.09 (30)
Withdrawal symptoms *	26.9 (31)

* Tolerance and withdrawal were excluded from the diagnosis criteria of prescribed -Opioid Use Disorder.

**Table 4:** A comparison of patients with moderate or severe prescribed-opioid use disorder (p-OUD) vs. no or mild p-OUD.

	No or Mild p-OUD (*N* = 42) % (n)	Moderate or Severe p-OUD (*N* = 32) %(*n*)	*p*
**Socio-professional variables**			
Gender (women) (%)	63.4 (26)	59.4 (19)	0.81
Age (years)	58.8	50.9	0.47
Sick leave	27.5 (11)	50.0 (15)	0.08
Pain duration (months)	102.5	117.7	0.35
Current treatment duration (months)	19.1	17.5	0.46
**Medical/psychiatric lifetime history (%)**			
Surgical (pain location)	65 (26)	40 (12)	0.05
Rheumatologic or orthopaedic	90 (36)	93.3 (28)	0.69
Cardiovascular	37.5 (15)	20 (6)	0.18
Digestive/urological/gynaecological	55.5 (22)	53.3 (16)	1
Neurological	17.5 (7)	16.6 (5)	1
Respiratory	22.5 (9)	20 (6)	1
Genetic/immunological/endocrine	17.5 (7)	20 (6)	1
Psychiatric	30 (12)	46.6 (14)	0.21
Addictions	5 (2)	20 (6)	0.06
**Current analgesics**			
**Non-opioid analgesics**	69.0 (29)	59.3 (19)	0.46
Paracetamol	95 (38)	93.3 (28)	1
Acetylsalicylic acid	10 (4)	10 (3)	1
Ibuprofen	57.5 (23)	66.6 (20)	0.46
**Weak PO**	78.5 (33)	68.7 (22)	0.42
Tramadol	65 (26)	70 (21)	0.79
Paracetamol + codeine	52.5 (21)	40 (12)	0.34
**Strong PO**	38.1 (16)	46.8 (15)	0.48
Skenan or actiskenan	22.5 (9)	40 (12)	0.12
Oxynorm or oxycontin	40 (16)	66.6 (20)	0.03
Durogesic	5 (2)	13.3 (4)	0.39
**Current associated treatment**			
Neuroleptic	23.8(10)	34.3 (11)	0.43
Antidepressant	9.5 (4)	34.3 (11)	0.01
Anxiolytic	30.9 (13)	50 (16)	0.14
Hypnotic	19 (8)	34.3 (11)	0.18
**Past use of analgesics**			
Paracetamol, ibuprofen, acetylsalicylic acid	97.5 (39)	93.3 (28)	0.57
Weak PO: Codeine, tramadol	90 (36)	80 (24)	0.30
Strong PO: Morphine, oxycodone	50 (20)	76.6 (23)	**0.02**
**Past associated treatment**			
Neuroleptic	45 (18)	60 (18)	0.23
Antidepressant	42.5 (17)	50 (15)	0.63
Anxiolytic	20 (8)	32.2 (10)	0.27
Hypnotic	10 (4)	12.9 (4)	0.72
**Non-pharmacological pain treatment**			
Kinesitherapy	67.5 (27)	68.9 (20)	1
Psychotherapy	12.5 (5)	34.5 (10)	**0.04**
Acupuncture	22.5 (9)	34.4 (10)	0.29
Osteopathy	27.5 (11)	37.9 (11)	0.43
TENS	17.5 (7)	24.1 (7)	0.55
Hypnosis	10 (4)	27.6 (8)	0.10
Relaxation	17.5 (7)	31 (9)	0.25
**PO misuse variables**			
Higher doses than prescribed	11.9 (5)	28.1 (9)	0.13
More frequent doses than prescribed	7.1 (3)	25 (8)	**0.04**
Other route of administration	7.1 (3)	0 (0)	0.25
Desire for effects other than analgesic	11.9 (5)	25 (8)	0.21
Sharing PO	2.3 (1)	3.1 (1)	1
Use of PO obtained from others	9.5 (4)	3.1 (1)	0.38
**Feelings of being dependent on their PO**	21.4 (9)	56.2 (18)	**0.007**

**Table 5:** Factors associated with moderate or severe p-OUD according to the multivariate logistic regression model.

Variables	Adjusted OR [CI 95%]	*p*
Current antidepressant use	7.14 [1.65–39.8]	0.01
Current oxycodone use	6.17 [1.15–46.1]	0.04
More frequent doses than prescribed	5.36 [0.82–45]	0.09
Psychotherapy	4.35 [1.02–22]	0.05
Current strong opioid use	0.28 [1.02–22]	0.17
Feeling dependent	3.73 [1.03–14.7]	0.04

**Table 6:** Factors associated with p-OUD according to the principal component analysis.

Variables	Factor Loading		
	F1	F2	F3
History of neurological disorders	**0.44**	0.078	0.083
Past use of antipsychotics	**0.597**	−0.237	0.19
Current use of strong opioids	**0.806**	0.092	−0.045
Past use of strong opioids	**0.862**	0.091	0.125
Sick leave	0.301	**0.524**	−0.01
Hypnosis	−0.057	**0.074**	0.061
Psychotherapy	−0.077	**0.839**	−0.005
History of psychiatric disorders	0.166	−0.096	**0.749**
History of addictive disorders	−0.007	0.087	**0.797**
Current antidepressant use	−0.228	0.291	**0.332**

## Data Availability

Data are available on reasonable request to the corresponding author.

## References

[1] Wilson-Poe A.R., Moron J.A. (2018). The dynamic interaction between pain and opioid misuse. Br. J. Pharmacol..

[2] Julien N., Lacasse A., Labra O., Asselin H. (2018). Review of chronic non-cancer pain research among Aboriginal people in Canada. Int. J. Qual. Health Care.

[3] Shipton E.A., Shipton E.E., Shipton A.J. (2018). A Review of the Opioid Epidemic: What Do We Do About It?. Pain Ther..

[4] Reid K.J., Harker J., Bala M.M., Truyers C., Kellen E., Bekkering G.E., Kleijnen J. (2011). Epidemiology of chronic non-cancer pain in Europe: Narrative review of prevalence, pain treatments and pain impact. Curr. Med. Res. Opin..

[5] Bouhassira D., Lanteri-Minet M., Attal N., Laurent B., Touboul C. (2008). Prevalence of chronic pain with neuropathic characteristics in the general population. Pain.

[6] Voon P., Karamouzian M., Kerr T. (2017). Chronic pain and opioid misuse: A review of reviews. Subst. Abus. Treat. Prev. Policy.

[7] Vadivelu N., Kai A.M., Kodumudi V., Sramcik J., Kaye A.D. (2018). The Opioid Crisis: A Comprehensive Overview. Curr. Pain Headache Rep..

[8] Clark D.J., Schumacher M.A. (2017). America’s Opioid Epidemic: Supply and Demand Considerations. Anesth. Analg..

[9] Chang K.C., Wang J.D., Saxon A., Matthews A.G., Woody G., Hser Y.I. (2017). Causes of death and expected years of life lost among treated opioid-dependent individuals in the United States and Taiwan. Int. J. Drug Policy.

[10] Gallagher A.M., Leighton-Scott J., van Staa T.P. (2009). Utilization characteristics and treatment persistence in patients prescribed low-dose buprenorphine patches in primary care in the United Kingdom: A retrospective cohort study. Clin. Ther..

[11] Chenaf C., Kabore J.L., Delorme J., Pereira B., Mulliez A., Zenut M., Delage N., Ardid D., Eshaller A., Authier N. (2019). Prescription opioid analgesic use in France: Trends and impact on morbidity-mortality. Eur. J. Pain.

[12] Kuijpers T., van Middelkoop M., Rubinstein S.M., Ostelo R., Verhagen A., Koes B.W., Van Tulder M.W. (2011). A systematic review on the effectiveness of pharmacological interventions for chronic non-specific low-back pain. Eur. Spine J..

[13] Noble M., Treadwell J.R., Tregear S.J., Coates V.H., Wiffen P.J., Akafomo C., Sholles K.M., Chou R. (2010). Long-term opioid management for chronic noncancer pain. Cochrane Database Syst. Rev..

[14] Krebs E.E., Gravely A., Noorbaloochi S. (2018). Opioids vs. Nonopioids for Chronic Back, Hip, or Knee Pain-Reply. JAMA.

[15] Lee M., Silverman S.M., Hansen H., Patel V.B., Manchikanti L. (2011). A comprehensive review of opioid-induced hyperalgesia. Pain Physician.

[16] Manchikanti L., Fellows B., Ailinani H., Pampati V. (2010). Therapeutic use, abuse, and nonmedical use of opioids: A ten-year perspective. Pain Physician.

[17] Breivik H., Collett B., Ventafridda V., Cohen R., Gallacher D. (2006). Survey of chronic pain in Europe: Prevalence, impact on daily life, and treatment. Eur. J. Pain.

[18] Franklin G.M., Mai J., Wickizer T., Turner J.A., Fulton-Kehoe D., Grant L. (2005). Opioid dosing trends and mortality in Washington State workers’ compensation, 1996–2002. Am. J. Ind. Med..

[19] Cicero T.J., Wong G., Tian Y., Lynskey M., Todorov A., Isenberg K. (2009). Co-morbidity and utilization of medical services by pain patients receiving opioid medications: Data from an insurance claims database. Pain.

[20] Atluri S., Akbik H., Sudarshan G. (2012). Prevention of opioid abuse in chronic non-cancer pain: An algorithmic, evidence based approach. Pain Physician.

[21] Benyamin R., Trescot A.M., Datta S., Buenaventura R., Adlaka R., Sehgal N., Glaser S.E., Vallejo R. (2008). Opioid complications and side effects. Pain Physician.

[22] American Psychiatric Association (2013). Diagnostic and Statistical Manual of Mental Disorders.

[23] Eiden C., Ginies P., Nogue E., Damdjy Y., Picot M.C., Donnadieu-Rigole H., Peyrière H. (2019). High Prevalence of Misuse of Prescribed Opioid Analgesics in Patients with Chronic Non-Cancer Pain. J. Psychoact. Drugs.

[24] Knisely J.S., Wunsch M.J., Cropsey K.L., Campbell E.D. (2008). Prescription Opioid Misuse Index: A brief questionnaire to assess misuse. J. Subst. Abus. Treat..

[25] Landreat M.G., Vigneau C.V., Hardouin J.B., Bronnec M.G., Marais M., Venisse J.L., Jolliet P. (2010). Can we say that seniors are addicted to benzodiazepines?. Subst. Use Misuse.

[26] Hasin D.S., O’Brien C.P., Auriacombe M., Borges G., Bucholz K., Budney A., Compton W.M., Crowley T., Ling W., Petry N.M. (2013). DSM-5 criteria for substance use disorders: Recommendations and rationale. Am. J. Psychiatry.

[27] Boscarino J.A., Rukstalis M.R., Hoffman S.N., Han J.J., Erlich P.M., Ross S., Gerhard G.S., Stewart W.F. (2011). Prevalence of prescription opioid-use disorder among chronic pain patients: Comparison of the DSM-5 vs. DSM-4 diagnostic criteria. J. Addict. Dis..

[28] Degenhardt L., Bruno R., Lintzeris N., Hall W., Nielsen S., Larance B., Cohen M., Campbell G. (2015). Agreement between definitions of pharmaceutical opioid use disorders and dependence in people taking opioids for chronic non-cancer pain (POINT): A cohort study. Lancet Psychiatry.

[29] Von Korff M., Walker R.L., Saunders K., Shortreed S.M., Thakral M., Parchman M., Hanse R.N., Ludman E., Sherman K.J., Dublin S. (2017). Prevalence of prescription opioid use disorder among chronic opioid therapy patients after health plan opioid dose and risk reduction initiatives. Int. J. Drug Policy.

[30] Boscarino J.A., Hoffman S.N., Han J.J. (2015). Opioid-use disorder among patients on long-term opioid therapy: Impact of final DSM-5 diagnostic criteria on prevalence and correlates. Subst. Abus. Rehabil..

[31] Just J.M., Schwerbrock F., Bleckwenn M., Schnakenberg R., Weckbecker K. (2019). Opioid use disorder in chronic non-cancer pain in Germany: A cross sectional study. BMJ Open.

[32] Edlund M.J., Martin B.C., Fan M.Y., Devries A., Braden J.B., Sullivan M.D. (2010). Risks for opioid abuse and dependence among recipients of chronic opioid therapy: Results from the TROUP study. Drug Alcohol Depend..

[33] Rolland B., Bouhassira D., Authier N., Auriacombe M., Martinez V., Polomeni P., Brousse G., Schwan R., Lack P., Bachelier J. (2017). Misuse and dependence on prescription opioids: Prevention, identification and treatment. Rev. Med. Interne.

[34] Moisset X., Trouvin A.P., Tran V.T., Authier N., Vergne-Salle P., Piano V., Martinez V. (2016). Utilisation des opioides forts dans la douleur chronique non cancéreuse chez l’adulte (SFETD). Presse Med..

[35] Butler S.F., Budman S.H., Fernandez K.C., Fanciullo G.J., Jamison R.N. (2009). Cross-Validation of a Screener to Predict Opioid Misuse in Chronic Pain Patients (SOAPP-R). J. Addict. Med..

[36] Butler S.F., Fernandez K., Benoit C., Budman S.H., Jamison R.N. (2008). Validation of the revised Screener and Opioid Assessment for Patients with Pain (SOAPP-R). J. Pain.

[37] Volkow N.D., Jones E.B., Einstein E.B., Wargo E.M. (2019). Prevention and Treatment of Opioid Misuse and Addiction: A Review. JAMA Psychiatry.

[38] Seidel S., Aigner M., Ossege M., Pernicka E., Wildner B., Sycha T. (2013). Antipsychotics for acute and chronic pain in adults. Cochrane Database Syst. Rev..

[39] Garland E.L., Brintz C.E., Hanley A.W., Roseen E.J., Atchley R.M., Gaylord S.A., Faurot K.R., Yaffe J., Fiander M., Keefe F.J. (2020). Mind-Body Therapies for Opioid-Treated Pain: A Systematic Review and Meta-analysis. JAMA Intern. Med..

[40] Chao Y.S., Ford C. (2019). Cognitive Behavioural Therapy for Chronic Non-Cancer Pain: A Review of Clinical Effectiveness.

[41] Day M.A., Ward L.C., Ehde D.M., Thorn B.E., Burns J., Barnier A., Mattingley B., Jensen M.P. (2019). A Pilot Randomized Controlled Trial Comparing Mindfulness Meditation, Cognitive Therapy, and Mindfulness-Based Cognitive Therapy for Chronic Low Back Pain. Pain Med..

[42] Williams R.M., Ehde D.M., Day M., Turner A.P., Hakimian S., Gertz K., Ciol M., McCall A., Kincaid C., Pettet M.W. (2020). The chronic pain skills study: Protocol for a randomized controlled trial comparing hypnosis, mindfulness meditation and pain education in Veterans. Contemp. Clin. Trials.

[43] Gueguen J., Barry C., Hassler C., Falissard B. (2015). Evaluation de l’effcacité de la pratique de l’hypnose. Inserm.

[44] Eilender P., Ketchen B., Maremmani I., Saenger M., Fareed A. (2016). Treatment approaches for patients with opioid use disorder and chronic noncancer pain: A literature review. Addict. Disord. Their Treat..

[45] Timmerman L., Stronks D.L., Groeneweg J.G., Huygen F.J. (2016). Prevalence and determinants of medication non-adherence in chronic pain patients: A systematic review. Acta Anaesthesiol. Scand..

[46] Chou R., Turner J.A., Devine E.B., Hansen R.N., Sullivan S.D., Blazina I., Dana T., Bougatsos C., Deyo R.I. (2015). The effectiveness and risks of long-term opioid therapy for chronic pain: A systematic review for a National Institutes of Health Pathways to Prevention Workshop. Ann. Intern. Med..

[47] Morasco B.J., Duckart J.P., Dobscha S.K. (2011). Adherence to clinical guidelines for opioid therapy for chronic pain in patients with substance use disorder. J. Gen. Intern. Med..

[48] Argoff C.E., Kahan M., Sellers E.M. (2014). Preventing and managing aberrant drug-related behavior in primary care: Systematic review of outcomes evidence. J. Opioid Manag..

[49] Kraus M., Lintzeris N., Bhaskar A., Alho H., Alon E., Bouhassira D., Haro G., D’Agnone O., Dematteis M., Kern K.-U. (2020). Consensus and Controversies Between Pain and Addiction Experts on the Prevention, Diagnosis, and Management of Prescription Opioid Use Disorder. J. Addict. Med..

[50] Von Korff M., Kolodny A., Deyo R.A., Chou R. (2011). Long-term opioid therapy reconsidered. Ann. Intern. Med..

[51] Ponton R., Sawyer R. (2018). Opioid prescribing in general practice: Use of a two-stage review tool to identify and assess high-dose prescribing. Br. J. Pain.

[52] Leach J.M. (2018). Managing addiction to prescribed opioids: The job of general practice?. Br. J. Gen. Pract..

[53] Shapiro H. Opioid Painkiller Dependency (OPD): An Overview. A Report Written for the All-Party Parliamentary Group on Prescribed Medicine Dependency 2015. https://www.drugsandalcohol.ie/25398/1/Opioid_painkiller_dependency_Sept_2015.pdf.

[54] Garcia-Orjuela M.G., Alarcon-Franco L., Sanchez-Fernandez J.C., Agudelo Y., Zuluaga A.F. (2016). Dependence to legally prescribed opioid analgesics in a university hospital in Medellin-Colombia: An observational study. BMC Pharmacol. Toxicol..

[55] Alho H., Dematteis M., Lembo D., Maremmani I., Roncero C., Somaini L. (2020). Opioid-related deaths in Europe: Strategies for a comprehensive approach to address a major public health concern. Int. J. Drug Policy.

[56] Kesten J.M., Thomas K., Scott L.J., Bache K., Hickman M., Campbell R., Pickering A.E., Redwood S. (2020). Acceptability of a primary care-based opioid and pain review service: A mixed-methods evaluation in England. Br. J. Gen. Pract..

